# Supplementary material for: Clinical, immune and genetic risk factors of malaria-associated acute kidney injury in Zambian children: A study protocol
Source: PLoS One. 2025 Feb 6;20(2):e0316205. doi: 10.1371/journal.pone.0316205 (PMC11801570; doi:10.1371/journal.pone.0316205)
Supplement: S2 File — (DOCX) [file pone.0316205.s002.docx]

**S2 Table 1: List of primer sequences to be used in the determination of the single nucleotide polymorphisms.**

| Gene | Nt | Mutation | Primers (5′-3′)) | Product size (bp) |
| --- | --- | --- | --- | --- |
| IL-10 (57)  (rs1800896) | -1082 | G A | Forward: TAA GGC TTC TTT GGG AG  Reverse: TAA GGC TTC TTT GGG AA | 258 |
| IL-10  (rs1800871) | -819 | C T | Forward: GGCTTCCTACAGTACAGGCG  Reverse: TTGGGAAGGGGAAGTAGGGA | 509 |
| TNF-α  (rs1800629) | -308 | G A | Forward: AGG TTT TGA GGG GCA TTG  Reverse: AGG TTT TGA GGG GCA TGA | 184 |
| TNF*α*  rs361525 (58) | −238 | **A G** | Forward: AGCCAAGACTGAAACCAGCA  Reverse: GTGTGCCAACAACTGCCTTT | 696 |
| IFNAR1 (rs2843710) | **-576** | **C   G** | Forward: CAAAACAGCCTAGCGACTGC  Reverse: GCACTTCACCATCGCATTCC | 443 |
| IFNAR1 (rs2243594) | Intron 2 | **A G** | Forward: GCGATGAGTCTGTCGGGAAT  Reverse: GAAGCTGGAACACCCAGAAGA | 314 |
| HMOX1 (rs7285877) | Intron 2 | **C T** | Forward: GGCCCCTCCAGGAAGGAGAAT  Reverse: AGGAGAGGGGAACAGCTGAA | 112 |
| NOS2  (rs8078340) | -1659 | **C T** | Forward: GCTTTCCCATCCCACTCCTC  Reverse: GCAGTGAAGATGAAGCCCCA | 393 |
| TGF-Beta 2  rs4846478 | Intron | **C G** | Forward: GTGGCTGCTGCCAAAAGTAA  Reverse: GCGCCATTGCCTCATATTTGT | 850 |
| TLR4 (rs4986791) | T399I | **C T** | Forward: GGCCTGTGCAATTTGACCAT  Reverse: ACTTCGAGACTGGACAAGCC | 590 |
