# Supplementary material for: Clinical, immune and genetic risk factors of malaria-associated acute kidney injury in Zambian children: A study protocol
Source: PLoS One. 2025 Feb 6;20(2):e0316205. doi: 10.1371/journal.pone.0316205 (PMC11801570; doi:10.1371/journal.pone.0316205)
Supplement: Attachment — Submitted filename: Chisambo et al reveiw comments.docx [file pone.0316205.s003.docx]

Chisambo et al shares a study protocol for an ongoing study to understand the burden, and risk factors for malaria associated acute kidney injury (MAKI)in children in Zambia. AKI is of growing importance in children in malaria endemic countries, and is a risk factor mortality Research to understand the pathogenesis are urgently needed.

There are two major issues that need to to addresses

Throughout the write up , there are several grammatical errors and loose/careless use of terms that the authors should correct

Abstract

A

Consider changing “escalates the risk of mortality sixfold”to increases the risk

and

93 “its long-term repercussions include impaired cognitive function and chronic kidney disease

94 (CKD), a phenomenon previously believed to be rare “. It is not clear which phenomenon the author is referring to here- CKD? OR AKI – both have previously been believed to be rare in children.

Methods

For objective 1and 2, clarify if the study population is <16-year s or 6months to 16-years. Reason for cut off at 16 years not clear?

Malaria pathogenesis and risk of complications including AKI, haemoglobinuria, severe anaemia etc. varies with age. There is a recent rise in the prevalence of AKI among older children, and indeed of haemoglobinuria in children older than 4 years.

The main issue is the study design for aims 1 and 2. An unmatched case control study is not the appropriate design to delineate risk factors for MAKI. Reasons for not match by age are not clear – Enroll children ages 6 to 16 years – match them by age: cases: AKI and controls non-AKI – Or at least by age groups. Given that at least 25% of children with severe malaria will develop AKI, these numbers can easily be estimated and matched

Additionally, all children referred to the study site with a diagnosis of MAKI already made will also be recruited as cases. Please clarify why you should depend on a referral diagnosis for enrollment

Study variables:

Renal outcome as a secondary outcome – This is presented here for the first time, out of nowhere, and needs a clearer definition.

Further laboratory results on admission include HRPC

365 levels, - correct HRPC

What does biomolecule mean as mentioned in several sentences in the protocol?

The Control group will be further categorized into subgroups, including cerebral malaria,

390 severe anemia, and uncomplicated malaria-? Not sure the study will be powered to see any differences. Further, what is the rationale? Why will children with un complicated malaria be followed for 3 days?
