# Supplementary material for: Clinical, immune and genetic risk factors of malaria-associated acute kidney injury in Zambian children: A study protocol
Source: PLoS One. 2025 Feb 6;20(2):e0316205. doi: 10.1371/journal.pone.0316205 (PMC11801570; doi:10.1371/journal.pone.0316205)
Supplement: Attachment — Submitted filename: RESPONSE TO REVIEWERS PLOS ONE.docx [file pone.0316205.s004.docx]

Department of Paediatrics and Child Health,

University of Zambia Medical School,

Lusaka, Zambia

The Editor-in-Chief,

PLOS ONE Journal,

05-07-2024.

Dear Dr Conroy,

Thank you for considering our manuscript and for the feedback received thus far.

Please find below a point-by-point response to the editor^’^s and reviewers^’^ comments.

**Editor^’^s:**

| 1 | **Editor comment:** At present, the manuscript is organized as a proposal to an IRB or higher education committee and does not align with standard approaches to write up study protocols. For example, rather than presenting the null hypotheses, objectives, and sample size calculations as bullet points and separate sections, I encourage the authors to summarize the information and present it instead as hypotheses, and objectives, and present the sample size calculation that was used to decide the final sample size.  **Response:** Done |
| --- | --- |
| 2 | **Editor comment**: Major revisions in the structuring of the content will be required for further consideration with the additional details requested by the reviewers. Please review the criteria on the website for guidance on how to structure protocol submissions and review published protocols for examples on what information to include. These guidelines must be followed for further consideration.  **Response:** Done |
| 3 | **Editor comment**: While an observational study does not have specific checklists for submission like clinical trials or systematic reviews, I strongly encourage the authors to review the SPIRIT and PRISMA checklists for study protocols and the STROBE guidelines on presenting results for observations studies to understand requirements for presentation of information  **Response:** Done.  The STROBE checklist for case-control studies submitted alongside the manuscript |
| 4 | **Editor comment:** Please ensure that your manuscript meets PLOS ONE's style requirements, including those for file naming.  **Response**: Done |
| 5 | **Editor comment: Y**our ethics statement should only appear in the Methods section of your manuscript. If your ethics statement is written in any section besides the Methods, please delete it from any other section.  **Response:** Done |

**Reviewer #1^,^s**

| 1 | **Reviewer’s comment: Title:-** To have “risk factors” and “predictors” in the title seems redundant, would select one of them.  **Response:** Title changed to:  “Clinical, immune and genetic risk factors of malaria-associated acute kidney injury in Zambian Children: A study protocol” |
| --- | --- |
| 2 | **Reviewer’s comment**: Abstract: - In the methods, it states AKI will be defined using the 2012 KDIGO criteria. Is this inclusive of both urine output and creatinine? If urine – how will this be quantified in incontinent children without indwelling bladder catheters.  **Response**: The urine output criterion of KDIGO 2012 will not be used in this study because the children that constitute the study population are not routinely monitored for urine output unless they are admitted to PICU.  Protocol amended to show this. |
| 3 | **Reviewer’s Comment**: Abstract: It states serum creatinine will be collected on admission and then at 48 hours post recruitment, but diagnosis of AKI is up to 72 hours. Creatinine will then have to be collected at 72 hours after admission. Please clarify this  **Response:** creatinine collection occurs at recruitment within the first 24 hours after admission (day 1) and then again on Day 3 and then finally at either discharge or day 7 whichever comes sooner.  Protocol amended with clearer phrasing |
| 4 | **Reviewer’s comment: Introduction**: A modified renal angina index is mentioned in the background, but in the methods, this is not described. Please clarify.  **Response:** This is presented under data analysis for sub-study 2:  “Local risk factors as identified from the cohort using multivariate logistic regression will be incorporated into the RAI in order to test if this will improve performance. Youden’s index will be used to determine the cut-off point for the modified RAI and curve fitting techniques will be utilized as described in previous studies by Matsuura et al. (57, 58,59) The AUC of the modified RAI and the AUC obtained from the original RAI will be compared using the z-test (non-parametric).”  References:  57. Matsuura R, Srisawat N, Claure-Del Granado R, Doi K, Yoshida T, Nangaku M, et al. Use of the Renal Angina Index in Determining Acute Kidney Injury. Kidney Int Rep. 2018;3(3):677-83.  58. Matsuura R, Iwagami M, Moriya H, Ohtake T, Hamasaki Y, Nangaku M, et al. A Simple Scoring Method for Predicting the Low Risk of Persistent Acute Kidney Injury in Critically Ill Adult Patients. Sci Rep. 2020;10(1):5726.  59. Zulu C, Mwaba C, Wa Somwe S. The renal angina index accurately predicts low risk of developing severe acute kidney injury among children admitted to a low-resource pediatric intensive care unit. Ren Fail. 2023;45(2):2252095. doi: 10.1080/0886022X.2023.2252095. |
| 5 | **Reviewer’s comment:** Introduction: - On page 5, line 98, it states that the development of CKD and ESKD are life limiting because of the constraints in access to KRT. Is it possible that these constraints also exist because of the resources available for transplant? Please add something about this.  **Response:** sentence re-written:  The development of CKD and end-stage kidney disease (ESKD) in these patients, is life-limiting due to constraints in access to kidney replacement therapy and transplantation similar to other low-resource environments. (10) |
| 6 | **Reviewer’s comment:** Introduction: On line 98-100, it states genetic and immune risk factors – this is very specific and should be also stated this way in the abstract, and perhaps even modify the title accordingly.  **Response:** Title changed to:  “Clinical, immune and genetic risk factors of malaria-associated acute kidney injury in Zambian Children: A study protocol” |
| 7 | **Reviewer’s comment:** Introduction: Line 103 – what is meant by “asexual forms”  **Response:** sentence modified to read “asexual parasite forms(merozoites, schizonts)”  Sentence deleted based on reviewer comment number 8. |
| 8 | **Reviewer’s comment:** Introduction: Line 106 – this belongs in the methods.  **Response:** the sentence in line 106 i.e  “For this study MAKI will be defined as evidence of the presence of malaria asexual forms in a child meeting the KIDGO 2012 acute kidney injury (AKI) criteria.”  Has been deleted from introduction. |
| 9 | **Reviewer’s comment:** Introduction: Can the authors state why they think the risk factors for malaria associated AKI in Zambian children might be different from other countries in Africa?  **Response: W**e plan to examine clinical, immune and genetic risk factor of MAKI in Zambian children.  Social-economic conditions in a given geographical location may alter the importance of previously described clinical risk factors. E.g local community propensity to use traditional healers prior to presentation may affect importance of herbal medications as a risk for MAKI, speed of seeking conventional health care.  Local medical practices may differ, and this may affect MAKI risk e.g. use of intravenous fluids, access to gentamicin. Access to organ support may further alter patient mortality rates.  Also, malaria transmission intensity alters population level immunity to malaria and this in turn may alter age groups affected by particular malaria syndromes.  Genetic predisposition e.g. The importance of various single nucleotide polymorphisms (SNPs) in risk to severe malaria varies between various ethnic groups.  Genetic makeup and distribution of SNPs across sub-Saharan Africa is diverse and previous studies have demonstrated that SNPs found to confer either susceptibility or protection to malaria / severe malaria syndromes in one part of Africa are not necessarily important when tested in other populations on the continent.  Similarly, because the function of immune proteins such as cytokines is determined by genes, the aforementioned genetic diversity in SNPs may result in varying importance of these immune proteins in the pathogenesis of MAKI in different ethnic groups.  The points cited above suggest that we need multicenter studies that include multiple ethnicities and geographical areas to further establish/confirm potential risk factors in MAKI.  Manuscript amended to include this sentence:  Page 5Line 101: “The importance of any particular risk factor in any given population may be modulated by peculiarities in local medical practice, access to healthcare resources, social determinants of health, changing malaria transmission intensity patterns and the effect of genetic variability on the function of the various immune molecules that are involved in MAKI pathogenesis.” |
| 10 | **Reviewer Comment:** Introduction: - There are scattered sentences throughout the introduction of what the investigators intend to do. It would be helpful if this could be summated in a single few sentences.  **Response:** Done |
| 11 | **Reviewer’s comment**: Introduction: The overall introduction seems a bit disjointed with the introduction of a new topic with each paragraph. Perhaps a better approach would be to discuss the clinical charactertisics and how the renal angina index could be modified for better AKI prediction, and then enhance that prediction by also integrating patient specific genotypes and immune testing, as well as the biomarkers to refine the prediction of AKI.  **Response**: Introduction revised to try and improve the flow as suggested. |
| 12 | **Reviewer’s comment**: Methods: - In the study objectives, it states that they will use the renal angina index for AKI prediction. While it may be that the authors will show that its performance is not as good as in other critically ill children, I think they should also state that its modification with population specific predictors will lead to enhanced performance of AKI prediction.  **Response:** Done. Specific objective 4 modified to:  “To determine the utility of the Renal Angina Score in predicting malaria-associated AKI and access whether its modification with population specific predictors will lead to enhanced performance of AKI prediction” |
| 13 | **Reviewer’s comment**: methods: Please define what is meant by a level II mixed intensive care unit.  **Response:** Sentence re-written:    “The paediatrics department has a capacity of 70 beds with access to level II intensive care unit (ICU) that admits neonates, children and adults with both medical and surgical conditions.” |
| 14 | **Reviewer’s comment**: Methods: The authors should consider how the onset of AKI might have occurred for those who are referred to UTH-CH, and the implications of their testing.  **Response:** Noted. We are collecting information on clinical characteristics like presence of diarrhea, vomiting, use of herbal medications, dose and type of drugs administered at home or the referral center. If blood results from the referring clinic are available these will be examined as well |
| 15 | **Reviewer’s comment**: Methods: - It would be helpful to define a list of inclusion and exclusion up front. It was not realized until later that children less than 6 months will be excluded.  **Response:** Done |
| 16 | **Reviewer’s comment**: Methods: - On line 244, please specify whether these KDIGO criteria will include UOP – and referring to my comment in the intro, how this is handled in incontinent children with urine/stool mixes.  **Response**: The study population includes children whose urine output is not routinely monitored and unless they are admitted to PICU are not catheterized.  For this reason, the UOP KDIGO 2012 criterion will not be used |
| 17 | **Reviewer’s comment**: Methods: The inclusion/exclusion criteria should be moved up above the definitions.  **Response**: Done |
| 18 | **Reviewer’s comment**: Methods: There is a mix of tense in the methods section – some of this is described in past tense, and some in future tense, and this should be consistent.  **Response**: Done |
| 19 | **Reviewer’s comment**: Methods: There are different sample sizes for each of the sub-analyses. Please provide a summative planned recruitment number with sufficient power. How will the renal angina index sample be determined?  **Response: Done**  **For recruitment into the RAI study:** Any participant with normal creatinine at admission (does not meet KDIGO creatinine criteria), will be recruited into the sub-study 2 (RAI) study. Page 22 line 411-415  The details on sample size estimation have been placed in supplementary file 1:  Sample size for the RAI study was determined using the Cochrane formula:      Where n = the required sample size  Z = Normal Standard Deviation taken with a 95% Confidence Interval; set at 1.96.  d = margin of error (0.05)  p = prevalence of AKI in malaria  Assumptions made were a significance level of 0.05, a prevalence of AKI in children admitted with malaria of 0.30 and power of 80%.  Allowing for a 10% loss of data a total sample size of 180 children with malaria and no AKI at admission to the hospital, will need to be recruited into the RAI study. |
| s20 | **Reviewer’s comment**: Methods: Again, in the methods there is inconsistency with earlier statements on when AKI will be defined – 48 or 72 hours.  **Response:** Participants are enrolled within the first 24 hours post admission (day 1) and blood is tested again for creatinine 48 hours later (Day 3).  Protocol harmonized. |
| 21 | **Reviewer’s comment**: Methods: On line 297 and 298, this is the only area where it seems that only serum creatinine will be used for the definition.  **Response**: The AKI criteria used has been clarified and harmonized throughout the manuscript.  Because this is a general hospital population urine output is not routinely monitored. Thus, for this study the urine output criterion of the KIDGO 2012 will not be used to define AKI.  Only the KIDGO 2012 creatinine criterion is used |
| 22 | **Reviewer’s comment**: Methods: Will laboratory tests be standardized across all patients?  **Response**: Yes. Specimens for chemistry, immunology will be frozen at -80 C while genetic samples will be collected on dried blood spots. The tests will be performed at UTH at either the KS molecular laboratory or UNZA veterinary school genomics laboratory or the UTH chemistry laboratory respectively. |
| 23 | **Reviewer’s comment**: Methods: The renal outcome as the secondary outcome needs a little more clarity in its definition on line 343 on page 17. This is a bit confusing as written.  **Response: Amended to read**  **“**Serum creatinine will be collected at discharge or if the patient is still hospitalized on day 7, whichever comes sooner, in order to establish the renal outcome at day 7. Renal outcome will be noted as a secondary outcome. Outcome will be classified as normal if eGFR is > 120ml/1.73m2/min or abnormal for eGFR < X. The Schwartz formula will be used to estimate GFR. “ |
| 24 | **Reviewer’s comment**: Methods: The description of how the RAI will be modified to best suit the population is not described.  **Response:** The statement below is placed in the data analysis plan for sub-study 2  “Local risk factors as identified from the cohort using multivariate logistic regression will be incorporated into the RAI in order to test if this will improve performance. Youden’s index will be used to determine the cut-off point for the modified RAI and curve fitting techniques will be utilized as described in previous studies by Matsuura et al. (57, 58) The AUC of the modified RAI and the AUC obtained from the original RAI will be compared using the z-test (non-parametric).”  References:  57. Matsuura R, Srisawat N, Claure-Del Granado R, Doi K, Yoshida T, Nangaku M, et al. Use of the Renal Angina Index in Determining Acute Kidney Injury. Kidney Int Rep. 2018;3(3):677-83.  58. Matsuura R, Iwagami M, Moriya H, Ohtake T, Hamasaki Y, Nangaku M, et al. A Simple Scoring Method for Predicting the Low Risk of Persistent Acute Kidney Injury in Critically Ill Adult Patients. Sci Rep. 2020;10(1):5726.  **59.** Zulu C, Mwaba C, Wa Somwe S. The renal angina index accurately predicts low risk of developing severe acute kidney injury among children admitted to a low-resource pediatric intensive care unit. Ren Fail. 2023;45(2):2252095. doi: 10.1080/0886022X.2023.2252095. |
| 25 | **Reviewer’s comment:** Methods: NGAL measurement is in the blood? Since it is found in neutrophils, would expect this to be elevated in many patients, not just with AKI. Have the authors considered uNGAL. Same is true for KIM-1, both of which indicate tubular dysfunction and will be present in the urine in patients with AKI.  **Response**: Due to unreliability of getting urine collection and to avoid placing bladder catheters it was decided to use serum biomarker levels as has been done in several other studies.  From literature both urine and plasma can be used to measure NGAL and KIM-1  References:   1. Patschan D, Erfurt S, Oess S, Lauxmann M, Patschan S, Ritter O, Hoffmeister M. Biomarker-Based Prediction of Survival and Recovery of Kidney Function in Acute Kidney Injury. Kidney Blood Press Res. 2023;48(1):124-134. doi: 10.1159/000528633. Epub 2023 Feb 9. Erratum in: Kidney Blood Press Res. 2023 Apr 28;48(1):260. PMID: 36758525. 2. Zdziechowska M, Gluba-Brzózka A, Poliwczak AR, Franczyk B, Kidawa M, Zielinska M, Rysz J. Serum NGAL, KIM-1, IL-18, L-FABP: new biomarkers in the diagnostics of acute kidney injury (AKI) following invasive cardiology procedures. Int Urol Nephrol. 2020 Nov;52(11):2135-2143. doi: 10.1007/s11255-020-02530-x. Epub 2020 Jun 17. PMID: 32557377; PMCID: PMC7575457. 3. Sabbisetti VS, Waikar SS, Antoine DJ, Smiles A, Wang C, Ravisankar A, Ito K, Sharma S, Ramadesikan S, Lee M, Briskin R, De Jager PL, Ngo TT, Radlinski M, Dear JW, Park KB, Betensky R, Krolewski AS, Bonventre JV. Blood kidney injury molecule-1 is a biomarker of acute and chronic kidney injury and predicts progression to ESRD in type I diabetes. J Am Soc Nephrol. 2014 Oct;25(10):2177-86. doi: 10.1681/ASN.2013070758. Epub 2014 Jun 5. PMID: 24904085; PMCID: PMC4178434. |
| 26 | **Reviewer’s comment**: methods: The way in which each of the sections of the methods are defined should follow the same order as earlier in the methods.  **Response:** Done |
| 27 | **Reviewer’s comment**: Methods: It is not clear why there are different patients being recruited for different portions of the study. If this is not true, there needs to be increased clarity on this.  **Response:** This has been clarified under the expanded study design section (Figure 1) |
| 28 | **Reviewer’s comment**: Methods: All the figures are blurry and cannot be commented on.  **Response:** All the figures have been re-worked |

**Reviewer #2**

| 1 | **Reviewer’s comment:** Throughout the write up, there are several grammatical errors and loose/careless use of terms that the authors should correct  **Response:** Done |
| --- | --- |
| 2 | **Reviewer’s comment**: Background: consider changing “escalates the risk of mortality six-fold” to increases the risk  **Response:** Done |
| 3 | **Reviewer’s Comment**: Background: 93 “its long-term repercussions include impaired cognitive function and chronic kidney disease  **Response:** Done |
| 4 | **Reviewer’s comment: Background**: 94 (CKD), a phenomenon previously believed to be rare “. It is not clear which phenomenon the author is referring to here- CKD? OR AKI – both have previously been believed to be rare in children.  **Response:** The phrase “a phenomenon previously believed to be rare “has been removed to avoid ambiguity |
| 5 | **Reviewer’s comment:** Methods: For objective 1and 2, clarify if the study population is <16-year s or 6months to 16-years. Reason for cut off at 16 years not clear?  **Response:** The inclusion criteria has been explained further.  Inclusion criteria: children between 6 months and 16 years who have parasitological evidence of malaria and who are admitted to either UTHs-CH or CCH. The two hospitals have an upper limit of 16 years to admission to paediatric services. |
| 6 | **Reviewer’s comment:** Methods: Malaria pathogenesis and risk of complications including AKI, haemoglobinuria, severe anaemia etc. varies with age. There is a recent rise in the prevalence of AKI among older children, and indeed of hemoglobinuria in children older than 5 years, and age is an important factor here that authors need to consider-- what if the majority of the cases are below five while controls are above 5?  **Response:** Agreed that the impact of age varies based on the local malaria transmission intensity with intermediate transmission areas reporting MAKI among younger patients whereas low unstable transmission areas report MAKI in older individuals. As malaria control programmes change the transmission landscape there are reports of changes in the pattern of AKI.  We have already recruited 100 children into the study without matching them at recruitment.  In view of the valid concerns raised we will match cases and controls based on age groups, less than 5 years, 5-10 years and greater than 10 years during analysis for the correlation between MAKI and various immune molecules such as cytokines and adhesion molecules.  We plan to test for immune molecules in only 200(100 cases and 100 controls) out of the total of 380 children that are being recruited. Thus, we will have sufficient numbers to select from during analysis of cytokines, adhesion molecules such that cases and controls are matched based on the aforementioned age groups.  The protocol has been amended accordingly Page 13-14:  “This is an exploratory study therefore no matching of cases to controls will be conducted at recruitment. However, during laboratory analysis of the various immune molecules 100 cases will be matched to 100 controls based on age groups (less than 5 years, 5-10 years and greater than 10 years) in order to account for the well-known effect of age on immune molecule serum concentrations.” |
| 7 | **Reviewer’s comment:** methods: The main issue is the study design for aims 1 and 2. An unmatched case control study is not the appropriate design to delineate risk factors for MAKI. Reasons for not match by age are not clear – Enroll children ages 6 to 16 years – match them by age: cases: AKI and controls non-AKI – Or at least by age groups. Given that at least 20-50% of children with severe malaria will develop AKI, these numbers can easily be estimated and matched  **Response:** Agreed that the impact of age varies based on the local malaria transmission intensity with intermediate transmission areas reporting MAKI among younger patients whereas low unstable transmission areas report MAKI in older individuals. As malaria control programmes change the transmission landscape there are reports of changes in the pattern of AKI.  We have already recruited 100 children into the study without matching them at recruitment.  In view of the valid concerns raised we will match cases and controls based on age groups, less than 5 years, 5-10 years and greater than 10 years during analysis for the correlation between MAKI and various immune molecules such as cytokines and adhesion molecules.  We plan to test for immune molecules in only 200(100 cases and 100 controls) out of the total of 380 children that are being recruited. Thus, we will have sufficient numbers to select from during analysis of cytokines, adhesion molecules such that cases and controls are matched based on the aforementioned age groups.  The protocol has been amended accordingly:  ““This is an exploratory study therefore no matching of cases to controls will be conducted at recruitment. However, during laboratory analysis of the various immune molecules 100 cases will be matched to 100 controls based on age groups (less than 5 years, 5-10 years and greater than 10 years) in order to account for the well-known effect of age on immune molecule serum concentrations.” |
| 8 | **Reviewer’s comment:** Methods: Additionally, all children referred to the study site with a diagnosis of MAKI already made will also be recruited as cases. Please clarify why you should depend on a referral diagnosis for enrollment  **Response:** RDT, serum creatinine will be repeated to confirm the diagnosis. |
| 9 | **Reviewer’s comment:** Methods: Study variables: Renal outcome as a secondary outcome – This is presented here for the first time, out of nowhere, and needs a clearer definition.  **Response: Amended to read**  **“**Serum creatinine will be collected at discharge or if the patient is still hospitalized on day 7, whichever comes sooner, in order to establish the renal outcome at Day 7. Renal outcome will be noted as a secondary outcome. Outcome will be classified as normal if eGFR is > 120ml/1.73m2/min or abnormal for eGFR < X. The Schwartz formula will be used to estimate GFR. “ |
| 10 | **Reviewer’s comment:** Methods: Study variables: Detailed description of hydration status, fluid balances with inputs and out puts, are important for MAKI. Further, suggest description of medicines used - e.g renal toxic medications to be collected between the groups as these are a potential but common risk factor for MAKI. How will severity for MAKI be categorized?  **Response:**  1. line 351-352 :The WHO classification for hydration status (classified according to WHO as none, some, and severe) will be used.  A reference to the WHO guideline has been added.  **2.** We will also document the serum urea to creatinine ratio  **3.** line 356-357 “Other clinical variables comprise duration of illness before admission, pre-admission medications and dosages…”  The particular variable on medication exposure was left open ended to so as to document all drugs and their dosages.   4. Inputs and outputs prior to arrival at the hospital are not available and for most malaria patients’ fluid input/outputs are hardly done unless there are specific concerns for renal dysfunction or in malaria patients that are admitted to PICU. 5. MAKI severity is classified based on KDIGO 2012 AKI classification as:   Stage 1, stage 2 and stage 3.  In contrast, in sub-study 2 where the RAI is investigated, AKI is grouped as non-severe AKI (KDIGO stage 1) and severe AKI (stage 2 and 3), similar to previous studies on RAI. |
| 11 | **Reviewer’s comment:** Methods: Study variables: Further laboratory results on admission include HRPC  365 levels, - correct HRPC  **Response: corrected to:**  “Plasmodium histidine rich protein 2 (PfHRP2)” |
| 12 | **Reviewer’s comment:** Methods: Study variables: What does biomolecule mean as mentioned in several sentences in the protocol?  **Response:** The word has been deleted from manuscript.  Instead “immune molecule” is used |
| 13 | **Reviewer’s comment:** Methods: Use of NGAL for as a biomarker of kidney injury is commendable  **Response:** Thank you |
| 14 | **Reviewer’s comment:** Methods: The Control group will be further categorized into subgroups, including cerebral malaria, 390 severe anemia, and uncomplicated malaria-? Not sure the study will be powered to see any differences. Further, what is the rationale? Why will children with uncomplicated malaria be followed for 3 days? What is the rationale for sun categorizing into CM, SMA but not other severe malaria groups? How will these groups be defined  **Response:**   1. The groups would be defined using the WHO severe malaria criteria (Severe malaria. Trop Med Int Health. 2014;19 Suppl 1:7-131.) 2. In view of concerns raised over study numbers being inadequate to power this, the sub-analysis will not be done. Protocol amended in line with this. |

Sincerely,

Chisambo Mwaba, BSc (Human Biology), MBChB, Mmed, Cert. Paed. Neph. (SA), MPhil

Lecturer

University of Zambia Medical School
