# Supplementary material for: Clinical, immune and genetic risk factors of malaria-associated acute kidney injury in Zambian children: A study protocol
Source: PLoS One. 2025 Feb 6;20(2):e0316205. doi: 10.1371/journal.pone.0316205 (PMC11801570; doi:10.1371/journal.pone.0316205)
Supplement: Attachment — Submitted filename: response to reviewers2.docx [file pone.0316205.s005.docx]

Department of Paediatrics and Child Health,

University of Zambia Medical School,

Lusaka, Zambia

The Editor-in-Chief,

PLOS ONE Journal,

03-10-2024.

Dear Dr Conroy,

Thank you for the feedback.

Please find below a point-by-point response to the editor^’^s and reviewers^’^ comments.

**Editor^’^s:**

| 1 | **Editor comment:** Thank you for your attention to the reviewers’ comments and work in revising the protocol. Both reviewers recognized the improvements in the manuscript but felt like there were still issues with the writing that needed to be addressed and some bigger picture inconsistencies regarding laboratory measures between sections of the protocol. As this study is currently recruiting, I strongly recommend that you use this feedback and critically review the protocol in detail to ensure each section is internally consistent and matches what is happening on the ground with recruitment. This will be an important resource for others looking to validate your work and will help with the ultimate write up and publication of the study findings.  **Response: Done** |
| --- | --- |
| 2 | **Editor comment**: I have reviewed the comments from the reviewers and consider all of their comments appropriate. Please address them point-by-point.  **Response:** Done |
| 3. | **Editor comment**: Provide an overview of what changes were made to ensure each section of the protocol is consistent.  **Response:** The language between the abstract and the methods section were harmonized. Throughout we now use “day” instead of hours to describe points at which serum creatinine will be collected in order to be consistent. Figure 1 further clarifies recruitment procedures.  The abstract has been revised to show that Day 7/discharge bloods are also being collected and that the study includes a nested prospective observational study.  Additionally, we added guidepost sentences such as:  - on page 12, line 256:  “In the following sections the recruitment and laboratory procedures, the study variables, and the data analysis plans for sub-study 1 (section 2.6) and the nested prospective sub-study 2 (section 2.7) have outlined.”  -page 8, line 179:  “The project is divided into two sub-studies, namely 1) Sub-study 1 which answers objectives 1, 2 and 3, and is an unmatched case-control study with a case:control ratio of 1:1. An unmatched design was chosen because this is an exploratory study. However, during analysis of data for the immune molecules, patients will be analysed based on age groups. Sub-study 2 answers objectives 4 and 5 and is a nested prospective observational study that recruits participants from the control arm of sub-study 1 (Fig1).“ |

**Reviewer #1^,^s**

| 1 | **Reviewer’s comment:** Overall: The protocol in its revised version is improved, although there are still many areas that are confusing and require revision for improved clarity. There continues to be inconsistency in when some laboratory measures will be obtained, varying between the abstract and body of the protocol.  **Response:** protocol harmonized |
| --- | --- |
| 2 | **Reviewer’s comment**: Title: Much improved, no further comments  **Response**: Thank you |
| 3 | **Reviewer’s Comment**: Abstract: In the responses, the authors state creatinine will also be measured on day 7 or discharge, whichever is sooner, but this is not mentioned in the section on collection of other creatinine tests.  **Response:** The statement below added to abstract: Page 4, line 60-64:  “AKI is defined using the 2012 KIDGO AKI creatinine criteria, and cases are defined as children admitted with malaria who develop AKI within 72 hours of admission, while controls are children admitted with malaria but with no AKI. Serum creatinine is collected on Day 1 within 24 hours of admission, on Day 3 and then again on discharge or day 7, whichever comes sooner. ” |
| 4 | **Reviewer’s comment: Abstract**: The utilization of the renal angina index should precede the brief description of statistical analysis  **Response:** the statement has been modified to read:  “The sensitivity, specificity, and estimates of the area under the curve (AUC) for the renal angina score will be determined.” |
| 5 | **Reviewer’s comment:** Introduction: This is substantially more succinct and clear that this will be a study protocol, and provides a construct for what is to come.  **Response:** Thank you |
| 6 | **Reviewer’s comment: Introduction:** Line 99 – this is a very long sentence that’s starts and ends with MAKI. Please revise for improved grammar separating risk factors and pathogenesis.  **Response: page 5: line 92-94:**  **‘**Risk factors in MAKI can be categorized as either biological or clinical. Biological risk factors are the various molecules involved in MAKI pathogenetic mechanisms such as cytoadherence, immune dysregulation, heme nephrotoxicity, and increased oxidative stress.[6, 7, 11, 12, 13] “ |
| 7 | **Reviewer’s comment: Introduction:** - Line 131 – the word cytoadherence is present 3 times in this sentence, please revise for improved grammar.  **Response: line 124-127: revised to read:**  Furthermore, cytoadherence causes endothelial activation, which in turn causes cytoadherence to worsen. Thus, a vicious cycle is established in which endothelial activation results from cytoadherence and vice versa. [11, 13, 25] |
| 8 | **Reviewer’s comment: Introduction:** In the responses, there are multiple references provided for the renal angina index. This modification for malaria and in low resource settings should be included and cited in the introduction.  **Response:** This sentence added to introduction: page 7: line 144-145  **“**Some studies have attempted to modify the RAI for use in different populations. [40,41,42]” |
| 9 | **Reviewer’s comment: Introduction:** - Line 158 (revised version) AKI and MAKI are in the same sentence, please revise for improved grammar.  **Response:** Sentence modified to read:  **“**We hypothesize that various clinical factors, serum concentration of immunological molecules, as well as genetic polymorphisms of selected immune factors are correlated to the risk of developing AKI in children admitted with malaria and can therefore be used for risk prediction.” |
| 10 | **Reviewer Comment:** Introduction: Hypothesis 3 states renal angina – is this the original one, or a modified version, please be specific and reference which is to be used.  **Response:** At this point we have not yet derived the parameters for the modified RAI as that will only be done once the data has been collected and regression analysis done.  Sentence modified to read: page 8: line 165:  “3) that the Renal Angina Index and/or its modification, NGAL, suPAR and KIM-1 when used in severe malaria may be useful in predicting severe AKI two days later (Day 3)” |
| 11 | **Reviewer’s comment**: Introduction: In aim 3, it states the biomarkers will be measured at 48 hours – but earlier, and in the figure it states day 3. This should be consistent.  **Response**: modified to:  “3) that the Renal Angina Index and/or its modification, NGAL, suPAR and KIM-1 when used in severe malaria may be useful in predicting severe AKI two days later (Day 3)” |
| 12 | **Reviewer’s comment**: Methods: Line 205 of when malaria occurs, perhaps this fits better in the introduction.  **Response:** sentence moved to section on study limitations |
| 13 | **Reviewer’s comment**: Methods: The sentence on line 209 “the paediatric department has a capacity…” is repeated, please correct.  **Response: Done** |
| 14 | **Reviewer’s comment**: Methods: - The methods section is still a bit confusing to follow and could be improved.  **Response:** Done. Added guidepost sentences such as:  - on page 12, line 256:  “In the following sections the recruitment and laboratory procedures, the study variables, and the data analysis plans for sub-study 1 (section 2.6) and the nested prospective sub-study 2 (section 2.7) are outlined.”  -page 8, line 179:  “The project is divided into two sub-studies, namely 1) Sub-study 1 which answers objectives 1, 2 and 3, and is an unmatched case-control study with a case:control ratio of 1:1. An unmatched design was chosen because this is an exploratory study. However, during analysis of data for the immune molecules, patients will be analysed based on age groups. Sub-study 2 answers objectives 4 and 5 and is a nested prospective observational study that recruits participants from the control arm of sub-study 1 (Fig1).“ |
| 15 | **Reviewer’s comment**: Methods: Line 355 – the Schwartz formula should be referenced. Is this the standard one with varying factors, or the modified bedside schwartz?  **Response:** Bedside. Reference provided:  56. Schwartz GJ, Haycock GB, Edelmann CM, Jr., Spitzer A. A simple estimate of glomerular filtration rate in children derived from body length and plasma creatinine. Pediatrics. 1976;58(2):259-63. |

**Reviewer #2**

| 1 | **Reviewer’s comment:** The authors have made great progress in revising the manuscript and have responded to most of the reviewer comments. There are scattered grammatical errors throughout the manuscript, including spacing, misplaced comas and spellings that need to be corrected before publication. The authors should take a keen look at the manuscript and correct all the errors.  **Response:** Grammatical errors corrected. |
| --- | --- |

Sincerely,

Chisambo Mwaba, BSc (Human Biology), MBChB, Mmed, Cert. Paed. Neph. (SA), MPhil

Lecturer

University of Zambia Medical School
